# Mental Toughness of Australian Army Recruits Undertaking Basic Military Training

**DOI:** 10.1093/milmed/usaf574

**Published:** 2025-11-30

**Authors:** Penelope Kitchingman, Grace Redden, Jace R Drain, Neil Gibson, John Sampson, Gregory Peoples, Christian Swann, Neanne Bennett, Herbert Groeller

**Affiliations:** School of Medical, Indigenous and Health Sciences, Faculty of Science, Medicine and Health, University of Wollongong, Wollongong, NSW 2522, Australia; School of Allied Health, Exercise and Sports Sciences, Faculty of Science and Health, Charles Sturt University, Bathurst, NSW 2795, Australia; School of Medical, Indigenous and Health Sciences, Faculty of Science, Medicine and Health, University of Wollongong, Wollongong, NSW 2522, Australia; Defence Science and Technology Group, Fishermans Bend, VIC 3207, Australia; School of Medical, Indigenous and Health Sciences, Faculty of Science, Medicine and Health, University of Wollongong, Wollongong, NSW 2522, Australia; School of Medical, Indigenous and Health Sciences, Faculty of Science, Medicine and Health, University of Wollongong, Wollongong, NSW 2522, Australia; Graduate School of Medicine, Faculty of Science Medicine and Health, University of Wollongong, Wollongong, NSW 2522, Australia; Physical Activity, Sport and Exercise Research Theme, Faculty of Health, Southern Cross University, Coffs Harbour, NSW 2450, Australia; Australian Defence Force, Canberra, ACT 2612, Australia; School of Medical, Indigenous and Health Sciences, Faculty of Science, Medicine and Health, University of Wollongong, Wollongong, NSW 2522, Australia

## Abstract

**Introduction:**

Military personnel require higher mental toughness to cope with volatile, uncertain, complex, and ambiguous environments. Recruit mental toughness was assessed before and after basic military training (BMT) to determine whether there are differences in mental toughness between recruits allocated to Combat Arms or All Corps, and whether BMT provides a setting conducive to altering recruit mental toughness.

**Materials and Methods:**

Three hundred and fifteen recruits (males: *n *= 270, females: *n *= 45) volunteered to participate in this study. Recruits were grouped according to their allocated Army employment category: Combat Arms or All Corps. The mental toughness questionnaire 48 was administered to recruits in week 1 (Pre) and week 12 (Post) of BMT. Body mass, aerobic fitness, and absolute and relative lower body strength were recorded in week 1 and week 8, aligning with the physical training schedule.

**Results:**

On-time completion of training was attained in 79% of all recruits and 21% were classified as off pathway (i.e., delayed or discharged). At Pre, Combat Arms recorded higher predicted VO_2max_ (3.9 mL^−1^·kg^−1^·min^−1^), absolute (37 kgf) and relative (0.5 kgf^−1^ˑBM^−1^) isometric mid-thigh pull, and Confidence (Interpersonal) (0.8) values compared with All Corps (*p *< 0.05). For recruits who completed BMT, there were no between-group differences for changes in mental toughness (*p *> 0.05). Instead, there was a mean decline in recruit global mental toughness (−5) following BMT, because of declines in dimensions: Commitment (−2.6), Confidence (Abilities) (−1.1), Control (Life) (−0.9) and Control (Emotion) (−0.7) (*p *< 0.05).

**Conclusions:**

Enlisted Army employment category (Combat Arms or All Corps), initial mental toughness and physical fitness did not influence changes in recruit mental toughness following BMT. Instead, BMT was associated with a mean decline in global mental toughness because of a decrease in several subscales linked to motivation, sense of control, and self-belief.

Key Messages

- Pre-enlistment Corps allocation, and starting physical fitness or strength are not associated with recruit mental toughness.
- Mean global mental toughness declines during basic military training among recruits.
- Declines in Commitment, Confidence (Abilities), Control (Emotion, Life) suggests a reduction in motivation, sense of control and self-belief among recruits enlisted in basic military training.

## INTRODUCTION

Within the Australian Army there are two overarching workforce segments, Combat Arms and All Corps. Of these two workforce segments, Combat Arms roles are the most physically and operationally demanding. To meet these elevated demands soldiers in combat roles require significantly higher levels of physical fitness compared to those soldiers serving non-combat duties.^1^ Physical activity and fitness are associated with mental toughness, with regularly active young adults and elite athletes obtaining higher scores on mental toughness tools compared to less active and nonathletic peers.^2^^,^^3^ Within the Australian Army, allocation of recruits to a Corp is determined at pre-enlistment before commencement of basic military training (BMT). Thus, many enlistees are unlikely to have a comprehensive understanding of demands or stressors associated with their assigned military trade when starting BMT. Therefore, our first aim in the investigation was to determine if the inherent attributes of superior physical fitness in recruits allocated to the Combat Arms Corp, afford recruits with higher scores in mental toughness before starting BMT.

An essential role of BMT is to prepare recruits for physically demanding duties encountered during both peacetime and operational settings.^1^ These environments are volatile, uncertain, complex, and ambiguous requiring not only superior physical fitness but also mental toughness to overcome setbacks so performance and combat effectiveness can be maintained.^1^ Mental toughness is a trait considered to reflect the strength of mind needed to achieve success and is often associated with high performance sporting environments.^4^^,^^5^ Within a military context, mental toughness has been described as an individual’s ability to persevere, overcome obstacles, and achieve their goal.^6^ A positive association between mental toughness and successful completion of the Australian Army Special Forces selection course has been reported earlier.^7^^,^^8^ However, little is known about the importance of mental toughness for successful completion of BMT in the Australian Army. Basic training aims to develop individual military skills and requires adaptation across physical, psychological, cultural, and social domains as individuals adjust to the military environment and its associated occupational requirements.^9^ These requisite and time-compressed adaptations may help to elucidate if mental toughness is more likely a dispositional-trait or state-like concept.^8^ For example, observer-rated mental toughness improved in a group of British Army parachute regiment recruits following a psychological skills intervention embedded within the respective basic training course.^10^

The success of recruits undertaking BMT may be influenced by mental toughness,^11^^,^^12^ which appears to be a malleable construct^13^ that can be improved in response to both long-term physical training^14^ and psychological skills training.^10^ To help ensure military personnel have the ability to cope with occupational stressors, military organizations have targeted the enhancement of physiological and psychological resilience of the workforce, commencing during BMT.^1^^,^^12^^,^^15^ However, it is currently unknown if mental toughness is modifiable or if the components that contribute to mental toughness change after exposure to stressors associated with BMT. This article aimed to determine if differences in physical fitness in Australian Army recruits are associated with differences in mental toughness. Specifically, it was hypothesized that recruits who were identified for Combat Arms would have superior physical fitness and higher scores in mental toughness before BMT. In addition, this study also investigated if mental toughness or its components, are malleable in recruits that successfully complete BMT. We hypothesized that regardless of Corps, given the significant improvement in physical fitness and performance, recruit mental toughness scores will improve after exposure to BMT.

## METHODS

For this prospective observational cohort study, all recruits completed a standardized 12-week training syllabus which included military education and skills lessons, and physical training.^16^ A self-reported mental toughness tool was administered to recruits and collected by the research team (i.e., non-military staff) during a mandatory medical screening session in week 1 (Pre) and again during week 12 (Post) of BMT. Platoon instructors were not present at the time of questionnaire completion to mitigate potential recruit response bias when responding to questions about mental toughness. All recruits completed the mental toughness tool at Pre and Post to ensure consenting and non-consenting recruits were not identifiable by military staff. However, questionnaires completed by non-consenting recruits were removed and confidentially disposed of by a member of the research team. Physical fitness and body mass were assessed during scheduled physical training sessions conducted by Army instructors in week 1-2 (Pre), and week 8-9 (Post), with results provided to the research team post-session. Training outcome data were retrospectively collected to identify recruits that had completed BMT on-time or that were off-pathway (i.e., delayed or discharged).

Three hundred and fifteen recruits (males: *n *= 270, females: *n *= 45) volunteered to participate in this study. Recruits were from training platoons that commenced BMT at the Army Recruit Training Centre, Kapooka (Australia) between August and December 2019. Recruits were grouped according to their allocated Army employ,yment category: (1) *Combat Arms* (i.e., infantry, artillery, armored, and combat engineer trainees); and (2) *All Corps* (i.e., aviation, band, catering, dental, medical, ordinance, military police, transport, electrical/mechanical engineers, signals, and intelligence recruits) (**Table 1**). Study procedures were explained to recruits before they provided written informed consent. The study was approved by the Departments of Defence and Veterans’ Affairs Human Research Ethics Committee (#083-18).

**Table 1.** Descriptive Statistics for Combat Arms (*n* = 191) and All Corps (*n* = 124) Recruits Upon Commencement of Basic Military Training Presented as Mean ± SD, Unless Stated Otherwise

	Combat arms	All corps
**Age** (y)	21.9 ± 3.8	24.2 ± 6.2*
**Gender** (n)		
Females	4	41
Males	187	83
**Training Outcome** (n)		
On Time	148	100
Off Pathway	43	24
**Body mass** (kg)	79.9 ± 12.1	76.7 ± 12.6*
**Predicted VO_2max_** (mL^−1^·kg^−1^·min^−1^)	45.1 ± 5.5	41.2 ± 3.3*
**Isometric mid-thigh pull (**kgf**)**	257 ± 51.2	220 ± 45.9*
**Isometric mid-thigh pull** (kgf^−1^ˑBM^−1^)	3.2 ± 0.5	2.9 ± 0.5*

* Significant difference between Combat Arms and All Corps recruits (*p *< 0.05).

The Mental Toughness Questionnaire—48 (MTQ48) is a widely used, validated self-report tool which provides a multidimensional model to identify (1) the contribution of mental toughness dimensions to a global score and (2) opportunities for targeted interventions.^17^^,^^18^ The MTQ48 consists of 48 items and uses a 5-point Likert scale (1 = *strongly disagree* and 5 = *strongly agree*) to assess individual thoughts and feelings across six dimensions associated with mental toughness.^19^ Recruits were instructed to respond to each item honestly as a reflection of their typical behavior. Completed MTQ48 were scored according to Clough, Earle, and Sewell,^17^ which provides a score for six dimensions, including Challenge, Commitment, Control (Emotion, Life), Confidence (Abilities, Interpersonal). These dimensions were further tallied to provide a global MTQ48 score. Internal consistency of the MTQ48 for the overall scale has been reported at α = 0.90, and its dimensions reported at α = 0.71-0.91^20^ and test-retest coefficients have also been reported at 0.80-0.90 for the overall scale and dimensions.^21^

Physical performance assessments were undertaken in physical training attire consisting of running shoes, shorts, and t-shirt, and under supervision by Army physical training instructors to ensure tests were executed in a safe and consistent manner. Before each assessment, recruits undertook a standardized warm-up involving dynamic whole-body movements and were familiarized with testing procedures by Army physical training instructors. Recruit body mass was assessed without shoes on calibrated platform scales (Wedderburn WS701, Sydney). The multi-stage fitness test was used to assess cardiorespiratory fitness in which final level and shuttle attained were used to estimate maximal oxygen consumption (predicted VO_2max_).^22^ An isometric mid-thigh pull (IMTP) assessed lower body strength using calibrated platform scales (Wedderburn WS701, Sydney). Recruits positioned their feet hip width apart, bar at mid-thigh height, knees flexed (30°-45°) using an overhand grip just outside shoulder width although maintaining a neutral spine. Recruits performed three attempts, progressively applying greater force upon the bar to reach peak force (kgf) within ∼3-5 sec followed by a minimum 2-minute rest. Recruit absolute and relative best effort has been reported.

Comparisons of Pre MTQ48 results between Combat Arms and All Corps recruits were determined using unpaired t-tests with Welch’s correction to account for unequal sample size and variation. Pearson’s correlation coefficient (*r*) was used to determine the association of MTQ48 dimensions and global mental toughness with age and initial physical fitness. Employment category and training interaction effects were determined using a two-way (group×time) repeated-measures analysis of variance. Statistical significance was accepted at *p *≤ 0.05, with data reported as mean ± standard deviation (SD) or 95% confidence intervals, unless stated otherwise using SPSS v25 (SPSS Inc., USA) and Prism v10 (GraphPad, USA). Descriptive results are reported for sex and training outcome (i.e., on-time and off-pathway).

## RESULTS

Of the 315 recruits, 248 (78.7%) successfully completed BMT on-time and 67 recruits (delayed: *n* = 44, 14%; discharged: *n* = 20, 6.3%) were classified as off-pathway. Of the recruits who completed BMT (on-time and delayed, *n* = 292), 207 (71%) completed the MTQ48 in its entirety during week 1 (Pre) and week 12 of training (Post). The remaining 29% of recruits who completed BMT either returned an incomplete MTQ48 or were delayed beyond the data collection period. The Pre and Post results from recruits who completed BMT were analyzed to examine the second aim of this study comparing Combat Arms (*n *= 112) and All Corps (*n *= 95) changes in mental toughness during BMT.

All Corps were 2 years older (*p *= 0.003) than Combat Arms and had a higher proportion of female recruits (**Table 1**). For Combat Arms, predicted VO_2max_, absolute, and relative IMTP were 3.9 mL^−1^·kg^−1^·min^−1^, 37.0 kgf, and 0.5 kgf^−1^ˑBM^−1^ higher respectively compared to All Corps (*p *< 0.001; **Table 1**). Combat Arms recorded higher (*p *< 0.02) MTQ48 Confidence (Interpersonal) scores for Pre compared with All Corps (**Table 2**). However, no difference (*p *> 0.05) was observed between the two groups in global or other MTQ48 dimension scores. There was a weak positive association for Challenge (*p *< 0.05) with predicted VO_2max_ (*r *= 0.20) and absolute IMTP (*r *= 0.13); and for Confidence (Interpersonal) (*p *= 0.004) with predicted VO_2max_ (*r *= 0.16). Remaining MTQ48 scores had no association (*p *> 0.05) with recruit age (*r *= −0.07 to 0.02), predicted VO_2max_ (*r *= −0.01 to 0.05), absolute IMTP (*r *= −0.01 to 0.08), or relative IMTP (*r *= −0.01 to 0.05).

**Table 2.** Mental Toughness Questionnaire 48 (MTQ48) Total Scores (Average Response) for Recruits, Who Completed Basic Military Training, in Combat Arms (*n* = 191) and All Corps (*n* = 124)

	Pre BMT		Post BMT	
	All Corps	Combat Arms	All Corps	Combat Arms
Challenge	30.0 ± 3.5 (3.8)	30.8 ± 3.4 (3.8)	29.8 ± 3.5 (3.7)	30.6 ± 3.4 (3.8)
Commitment	42.2 ± 5.2 (3.9)	42.6 ± 5.6 (3.9)	39.3 ± 6.0 (3.6)	40.8 ± 6.0 (3.7)
Control (Emotion)	23.5 ± 3.1 (3.4)	24.1 ± 3.5 (3.5)	23.2 ± 3.5 (3.3)	23.5 ± 3.0 (3.4)
Control (Life)	24.6 ± 3.4 (3.5)	24.7 ± 3.6 (3.6)	23.6 ± 3.2 (3.4)	24.2 ± 3.1 (3.5)
Confidence (Abilities)	33.2 ± 4.3 (3.7)	32.9 ± 4.5 (3.7)	31.8 ± 4.6 (3.5)	32.4 ± 4.4 (3.6)
Confidence (Interpersonal)	20.7 ± 3.0 (3.5)	21.5 ± 3.4* (3.6)	20.9 ± 3.4 (3.5)	22.2 ± 3.2 (3.7)
**Global**	**174 ± 18.0 (3.6)**	**177 ± 19.6 (3.7)**	**168.7 ± 18.5 (3.5)**	**173.8 ± 17.6 (3.6)**

* Significantly different (*p *< 0.05) to All Corp. All data presented as mean ± SD.

For the recruits who completed BMT, Combat Arms and All Corps responded similarly, with no significant interactions observed in physical fitness (*p* = 0.18-0.43) or MTQ48 responses (*p* = 0.24-0.76, **Table 2**). The main effect of BMT over time demonstrated a decline in recruit body mass (−0.58 kg, *p *= 0.03) and increase in predicted VO_2max_ (2.9 mL^−1^·kg^−1^·min, *p *< 0.001), absolute IMTP (23.8 kgf, *p *< 0.001), and relative IMTP (0.4 kgf^−1^ˑBM^−1^, *p *< 0.001) (**Table 3**). Furthermore, global MTQ48 declined (−5.0, *p *= 0.002) Post BMT (**Table 3**). Commitment (−2.6, *p *= 0.002), Confidence (Abilities) (−1.1, *p *= 0.004), Control (Life) (−0.9, *p *= 0.002) and Control (Emotion) (−0.7, *p *< 0.001) dimensions declined after recruits completed BMT (**Table 3**).

**Table 3.** Change in Recruit Body Mass, Physical Fitness, and Mental Toughness Questionnaire 48 (MTQ48) Total Responses (Average Response) Following Completion of Basic Military Training (BMT; *n *= 207)

	Pre BMT	Post BMT	Change
**Body mass** (kg)	79.7 ± 11.9	79.1 ± 10.4	−0.6 (−0.04,−1.1)*
**Predicted VO_2max_** (mL^−1^·kg^−1^·min^−1^)	42.8 ± 4.4	45.7 ± 4.2	2.9 (2.4,3.2)*
**Isometric mid-thigh pull (**kgf**)**	242.0 ± 48.2	265.8 ± 44.5	23.8 (19.8,28.6)*
**Isometric mid-thigh pull** (kgf^-1^ˑBM^-1^)	3.0 ± 0.5	3.4 ± 0.4	0.4 (0.3,0.4)*
**MTQ48 Scores**			
**Challenge**	30.4 ± 3.2 (3.8)	30.2 ± 3.5 (3.8)	−0.2 (−0.6,0.3) (−)
**Commitment**	42.7 ± 5.0 (3.9)	40.1 ± 6.0 (3.6)	−2.6 (−3.7,−1.5)* (−0.3)
**Control** (Emotion)	24.1 ± 3.0 (3.4)	23.4 ± 3.2 (3.3)	−0.7 (−1.3,−0.1)* (−0.1)
**Control** (Life)	24.8 ± 3.2 (3.5)	23.9 ± 3.2 (3.5)	−0.9 (−1.5,−0.3)* (−0.1)
**Confidence** (Abilities)	33.3 ± 4.1 (3.7)	32.1 ± 4.5 (3.6)	−1.1 (−1.9,−0.4)* (−0.1)
**Confidence** (Interpersonal)	21.2 ± 3.1 (3.5)	21.6 ± 3.4 (3.6)	0.5 (−0.1, 1.0) (0.1)
**Global**	**176.4 ± 17.0 (3.7)**	**171.4 ± 18.1 (3.6)**	−5.0 (−8.1, −1.9)* (−0.1)

* Significant change from Pre BMT to Post BMT (*p *< 0.05) in all recruits. All data presented as mean ± SD or mean difference and confidence interval.

## DISCUSSION

The findings from this study identified a higher level of Confidence (Interpersonal) scores for recruits enlisted in Combat Arms employment categories at Pre compared with those enlisted in All Corps employment categories. However, of all recruits that completed the 12-week BMT (66%, *n* = 207) and graduated as military trainees, a significant decline in the mean global mental toughness score was observed, with dimensions of Commitment, Control (Emotion, Life) and Confidence (Abilities) predominantly contributing to the decrease. The lack of between-group differences for Combat Arms and All Corps in Pre and Post physical fitness and MTQ48 scores suggest that entry level fitness and pre-BMT employment category allocation is not indicative of how recruit mental toughness will change over BMT. This is perhaps because of the standardized military and physical fitness program delivered to all recruits during BMT before moving into employment-specific training. Furthermore, the decline in global score occurred despite improvements in recruit physical fitness (cardiovascular fitness and muscular strength), underscoring a lack of association between mental toughness and physical fitness in this investigation.

Contrary to our hypothesis, we observed a decline in global mental toughness following BMT regardless of allocated employment categories. Our results exclusively report recruits who completed a 12-week BMT program, thus, results are specific to this cohort only. Three dimensions, Commitment, Control (Emotion, Life), and Confidence (Abilities), accounted for the reported decline in global mental toughness. According to the 4Cs model,^17^ changes in these three dimensions suggest a reduction in recruit motivation to achieve goals, a loss of control, and reduced belief in one’s own ability. Previous findings have similarly reported feelings of disappointment, a sense of loss and lack of personal control because of pressures of adjusting to new standards of behavior and avoidance of aversive consequences when making the transition from civilian to military recruit.^23^ This finding is also consistent with research on the change in resilience and psychological well-being following enlistment. Basic training is associated with a period of adjustment and a mild increase in negative psychological symptoms, followed by an improvement as individuals move into employment specific training.^24^

Interestingly, mental toughness has been shown to improve during employment specific training,^10^ which follows BMT. A decrease in mental toughness during BMT may reflect a loss of autonomy as individuals adjust to a military environment where there is less freedom and opportunity to make their own choices and they are yet to develop a sense of competence or professional mastery.^23^^,^^25^^,^^26^ Lepinoy, Lo Bue, and Vanderlinde^27^ identified that when needs for autonomy, relatedness, and competence are met, optimal functioning is more likely to be achieved. Similar declines in individual effort, motivation, and sense of identity, have also been shown in sporting contexts.^28^ In addition, the potential for fatigue and poor sleep conditions during BMT may also contribute to a decline in mental toughness, with findings from a systematic review identifying poor sleep quality and quantity is related to lower levels of resilience.^29^ Further, a previous publication with a subset of this study’s cohort, suggested that 42% of BMT recruits experienced chronic sleep restriction during the 12-week program.^16^

A decline in mental toughness in BMT, as observed in this study, may not be predictive of long-term military success, particularly given evidence which suggests mental toughness increases after BMT.^10^ In addition, several studies have identified a positive association between mental toughness and successful completion of Special Forces selection.^7^^,^^8^ Taken together, our findings and those of previous military cohorts support the notion of mental toughness being a malleable construct that is sensitive to environmental factors.^18^ However, improvements in mental toughness may support reduced rates of attrition during BMT, with targeted mental health interventions for recruits at-risk of experiencing training delays or early discharge. Especially given that cognitive-behavioral upskilling demonstrates improved functioning and performance, reduced attrition, positive states of mind, and lower levels of distress throughout BMT.^30^^,^^31^ Such findings, in conjunction with the decline in global mental toughness observed in this study, support the idea of implementing tailored cognitive-behavioral upskilling incorporating military-specific content to provide opportunity for recruit resilience improvement. Thus, expediate improvements in mental toughness within the recruit training continuum and potentially mitigate attrition in the early stages of Army training.^32^

At commencement of BMT, Combat Arms recruits recorded statistically higher Confidence (Interpersonal) scores suggestive of a more assertive nature. However, the between-group difference in score was small (0.8 points) and there were no further differences for the remaining MTQ48 dimension scores. Together these findings indicate a lack of meaningful difference in mental toughness between Combat Arms and All Corps recruits at the beginning of BMT. Moreover, there was no association between mental toughness and the measured physical fitness variables, indicating that they may not be a strong attributor to mental toughness in military recruits despite the higher fitness observed for Combat Arms recruits at Pre. Cross-sectional investigations have shown that mental toughness in civilian populations typically increase with advancing expertise/participation in exercise and sporting environments.^2^^,^^3^ For example, Vaughan, Hanna, and Breslin^3^ presented mean global mental toughness results which were 44 points higher for elite athletes (182 points) compared with nonathletes (138 points). Likewise, global mental toughness incrementally increased among adolescents and young adults who participated in 1-7 days of moderate to vigorous physical activity compared with their nonactive counterparts.^2^ Therefore, fitness is unlikely to be the contributing factor to enhancing mental toughness. Instead, it is possible that the involvement in physical activity, exercise, and sport provide a suitable environment for the acquisition of mental toughness attributes.^2^ This is likely to reflect a sense of competence or skill mastery. In a military training context, this sense of competence is more likely to be experienced as recruits progress from BMT into employment specific training, potentially explaining why mental toughness increases during training downstream of BMT. In this study, recruits pre-enlisted into Combat Arms employment categories had higher levels of fitness compared with All Corps recruits which may reflect greater engagement in exercise and/or sport before the start of BMT. However, given the pre-enlistment fitness standards, the recruits of this study were a more homogenous group with respect to physical fitness compared with previous cross-sectional studies; likely explaining why differences in mental toughness were not extensive compared with those previously observed.^2^^,^^3^

The results of this article extend prior research by assessing self-reported multidimensional components of mental toughness among a military cohort. However, we acknowledge that the MTQ48 was developed using a different conceptualization of mental toughness compared with that of a military perspective. Further, given the MTQ48 is a self-report tool it is susceptible to response bias, particularly social desirability. Despite mitigating response bias by administering the MTQ48 in the absence of military staff, recruits may have still responded in a perceived favourable way potentially inflating Pre scores. Nevertheless, given the structured, consistent, and anonymous data collection procedures across time points, this risk of bias was minimized. We also did not measure recruit motivation for this study which may have provided further insight into the mean decline in global mental toughness score given that recent studies have shown that higher motivation for training tends to lead to greater performance.^33^ As such, future research applicable for military cohorts may consider resilience training programs which provide opportunities to sustain skills and interest among personnel for long-term success.

## CONCLUSION

In conclusion, there appears to be a global reduction in mental toughness score among Australian Army recruits following BMT, regardless of enlisted Corps, initial mental toughness, or fitness; unlike the physical improvements observed, for aerobic fitness and lower body strength. In conjunction with the lack of association between Pre mental toughness and initial fitness, these findings suggest there is an opportunity for targeted interventions, such as instructor-led mental toughness training.^12^ Such interventions should go beyond physical and military-skills training to better equip recruits for the unique emotionally and cognitively demanding environments associated with military service.

## Data Availability

The data that support the findings of this study are available on request from the corresponding author.

## INSTITUTIONAL CLEARANCE

Institutional clearance is approved.

## CLINICAL TRIAL REGISTRATION NUMBER

Nil.

## PRIOR PRESENTATION

Nil.

## References

[1] Nindl BC , Billing DC, Drain JR, et al Perspectives on resilience for military readiness and preparedness: report of an international military physiology roundtable. J Sci Med Sport. 2018;21:1116–24. 10.1016/j.jsams.2018.05.005 29886134

[2] Gerber M , Kalak N, Lemola S, et al Adolescents’ exercise and physical activity are associated with mental toughness. Ment Health Phys Act. 2012;5:35–42. 10.1016/j.mhpa.2012.02.004

[3] Vaughan R , Hanna D, Breslin G. Psychometric properties of the mental toughness questionnaire 48 (MTQ48) in elite, amateur and nonathletes. Sport Exerc Perform Psychol. 2018;7:128–40. 10.1037/spy0000114

[4] Connaughton D , Wadey R, Hanton S, Jones G. The development and maintenance of mental toughness: perceptions of elite performers. J Sports Sci. 2008;26:83–95. 10.1080/02640410701310958 17852671

[5] Crust L , Azadi K. Mental toughness and athletes’ use of psychological strategies. Eur J Sport Sci. 2010;10:43–51. 10.1080/17461390903049972

[6] Gucciardi DF , Lines RLJ, Ducker KJ, Peeling P, Chapman MT, Temby P. Mental toughness as a psychological determinant of behavioral perseverance in special forces selection. Sport Exerc Perform Psychol. 2021;10:164–75. 10.1037/spy0000208

[7] Gucciardi DF. Mental toughness: taking stock and considering new horizons. In: Tenenbaum G, Eklund RC, Boiangin N, eds. Handbook of Sport Psychology. 4th ed. John Wiley & Sons; 2020:101–120.

[8] Gucciardi DF , Hanton S, Gordon S, Mallett CJ, Temby P. The concept of mental toughness: tests of dimensionality, nomological network, and traitness. J Pers. 2015;83:26–44. 10.1111/jopy.12079 24428736

[9] Groeller H , Burley S, Orchard P, Sampson JA, Billing DC, Linnane D. How effective is initial military-specific training in the development of physical performance of soldiers? J Strength Cond Res. 2015;29:S158–62. 10.1519/jsc.0000000000001066 26506181

[10] Fitzwater JPJ , Arthur CA, Hardy L. “The tough get tougher”: mental skills training with elite military recruits. Sport Exerc Perform Psychol. 2018;7:93–107. 10.1037/spy0000101

[11] Beattie S , Du Preez T, Hardy LEW, Arthur C. What do you bring to the table? exploring psychological attributes that predict successful military training. *Mil Psychol*. 2023;37:50–61. 10.1080/08995605.2023.2285693 38015963 PMC11649220

[12] Saul KM , Young MD, Siddiqi JM, Hirsch DA. Developing a mental toughness program for basic military training. Mil Psychol. 2024;36:203–13. 10.1080/08995605.2023.2167467 38377247 PMC10890711

[13] Lin Y , Clough PJ, Welch J, Papageorgiou KA. Individual differences in mental toughness associate with academic performance and income. Pers Individ Differ. 2017;113:178–83. 10.1016/j.paid.2017.03.039

[14] Marshall T , Roberts J, Pack S, et al The effect of long term physical training on the development of mental toughness in recreationally active participants. J Multidiscip Res. 2017;9:29–43.

[15] Bartone PT. Resilience under military operational stress: can leaders influence hardiness? Military Psychology. 2006;18:S131–S148. 10.1207/s15327876mp1803s_10

[16] Larsen P , Drain JR, Gibson N, et al Chronicity of sleep restriction during army basic military training. J Sci Med Sport. 2022;25:432–8. 10.1016/j.jsams.2022.01.008 35277344

[17] Clough P , Earle K, Sewell D. Mental toughness: the concept and its measurement In: Cockerill I, ed. Solutions in Sport Psychology. Thomson Learning; 2002:32–43.

[18] Lin Y , Mutz J, Clough PJ, Papageorgiou KA. Mental toughness and individual differences in learning, educational and work performance, psychological well-being, and personality: a systematic review. Front Psychol. 2017;8:1345. 10.3389/fpsyg.2017.01345 28848466 PMC5554528

[19] Perry JL , Clough PJ, Crust L, Earle K, Nicholls AR. Factorial validity of the mental toughness questionnaire-48. Pers Individ Differ. 2013;54:587–92. 10.1016/j.paid.2012.11.020

[20] Nicholls AR , Polman RCJ, Levy AR, Backhouse SH. Mental toughness, optimism, pessimism, and coping among athletes. Pers Individ Differ. 2008;44:1182–92. 10.1016/j.paid.2007.11.011

[21] Clough P , Strycharczyk D. Developing Mental Toughness: Improving Performance, Wellbeing and Positive Behaviour in Others. Kogan Page; 2012.

[22] Ramsbottom R , Brewer J, Williams C. A progressive shuttle run test to estimate maximal oxygen uptake. Br J Sports Med. 1988;22:141–4. 10.1136/bjsm.22.4.141 3228681 PMC1478728

[23] Kiernan MD , Repper J, Arthur A. Why do they fail? A qualitative follow up study of 1000 recruits to the british army infantry to understand high levels of attrition. Work. 2015;52:921–34. 10.3233/wor-152208 26599676

[24] Dell L , Fredrickson J, Sadler N, et al The Longitudinal Australian Defence Force Study Evaluating Resilience (LASER): 2009–19—A Summary Report of the Research Program. Phoenix Australia; 2019.

[25] Bulmer S , Drain JR, Tait JL, et al Quantification of recruit training demands and subjective wellbeing during basic military training. Int J Environ Res Public Health. 2022;19:7360. 10.3390/ijerph19127360 35742608 PMC9223755

[26] Kaiser J , Buciuman M, Gigl S, Gentsch A, Schütz-Bosbach S. The interplay between affective processing and sense of agency during action regulation: a review. Front Psychol. 2021;12:716220. 10.3389/fpsyg.2021.716220 34603140 PMC8481378

[27] Lepinoy A , Lo Bue S, Vanderlinde R. Basic needs satisfaction in a military learning environment: an exploratory study. Mil Psychol. 2022;34:98–109. 10.1080/08995605.2021.1973793 38536337 PMC10013457

[28] Haugen T , Reinboth M, Hetlelid KJ, Peters DM, Høigaard R. Mental toughness moderates social loafing in cycle time-trial performance. Res Q Exerc Sport. 2016;87:305–10. 10.1080/02701367.2016.1149144 26958707

[29] Arora T , Grey I, Östlundh L, et al A systematic review and meta-analysis to assess the relationship between sleep duration/quality, mental toughness and resilience amongst healthy individuals. Sleep Med Rev. 2022;62:101593. 10.1016/j.smrv.2022.101593 35462348

[30] Williams A , Hagerty BM, Yousha SM, Horrocks J, Hoyle KS, Liu D. Psychosocial effects of the boot strap intervention in navy recruits. Mil Med. 2004;169:814–20. 10.7205/MILMED.169.10.814 15532347

[31] Cohn A , Pakenham K. Efficacy of a cognitive-behavioral program to improve psychological adjustment among soldiers in recruit training. Mil Med. 2008;173:1151–7. 10.7205/MILMED.173.12.1151 19149330

[32] Thompson MM , McCreary DR. Enhancing mental readiness in military personnel. Military Life: The Psychology of Serving in Peace and Combat. 2006;2:54–79.

[33] Niederhauser M , Zueger R, Sefidan S, Annen H, Brand S, Sadeghi-Bahmani D. Does training motivation influence resilience training outcome on chronic stress? Results from an interventional study. Int J Environ Res Public Health. 2022;19. 10.3390/ijerph19106179 PMC9140799 35627725

